# Association between Baltic sea diet and healthy Nordic diet index with risk of non-alcoholic fatty liver disease: a case–control study

**DOI:** 10.1038/s41598-024-60400-3

**Published:** 2024-04-25

**Authors:** Zahra Rasoulizadeh, Abolfazl Namazi, Mohammad Hassan Sohouli, Pejman Rohani, Azita Hekmatdoost, Mahdieh Hosseinzadeh

**Affiliations:** 1 School of Medicine, Shahid Sadoughi University of Medical Sciences, Yazd, Iran; 2 Department of Internal Medicine, Hazrat-E Rasool General Hospital, Iran University Of Medical Sciences, Tehran, Iran; 3 Student Research Committee, Department of Clinical Nutrition and Dietetics, Faculty of Nutrition and Food Technology, National Nutrition and Food Technology Research Institute, Shahid Beheshti University of Medical Sciences, No 7, West Arghavan St, Farahzadi Blvd, PO Box 19395-4741, Tehran, 1981619573 Iran; 4 Pediatric Gastroenterology and Hepatology Research Center, Pediatrics Centre of Excellence, Children′s Medical Center, Tehran University of Medical Sciences, Tehran, Iran; 5 Department of Clinical Nutrition and Dietetics, Faculty of Nutrition and Food Technology, Shahid Beheshti University of Medical Sciences, Tehran, Iran; 6 Department of Nutrition, School of Public Health, Shahid Sadoughi University of Medical Sciences, Yazd, Iran

**Keywords:** NAFLD, Nordic diet, Baltic sea diet, Chronic diseases, Case–control, Endocrinology, Gastroenterology, Risk factors

## Abstract

Recent evidence shows the beneficial effects of Baltic Sea diet score (BSDS) and healthy Nordic diet index (HNDI) on chronic diseases, however, there is no evidence to investigate them on the risk of non-alcoholic fatty liver disease (NAFLD). The purpose of this study was to investigate the associations between BSDS and HNDI with the risk of NAFLD. In this case–control study, 552 people in good health and 340 people with NAFLD over the age of 18 took part. The evaluation of BSDS and HNDI employed a validated 168-item semi-quantitative food frequency questionnaire (FFQ). Binary logistic regression was used to determine how OBS and NAFLD are related. The mean BSDS and HNDI were 16.00 ± 2.49 and 11.99 ± 2.61, respectively. The final model's confounder adjustment revealed that greater HNDI adherence scores gave protection against the occurrence of NAFLD (odds ratio [OR]: 0.42; 95% confidence interval [CI] 0.18–0.98; P for trend = 0.043). In addition, those with the highest BSDS scores had significantly lower risks of developing NAFLD compared to subjects with the lowest scores (OR = 0.48, 95% CI 0.32–0.89; p for trend = 0.003). Our findings showed that following a healthy Nordic diet can significantly prevent the risk of developing NAFLD, and suggest that the highly nutritious components of the Nordic diet are beneficial for the prevention of NAFLD.

## Introduction

The most prevalent kind of chronic liver disease is non-alcoholic fatty liver disease (NAFLD), which is characterized by a variety of fat liver conditions that can lead to cirrhosis and severe liver disease^1^. Adult NAFLD prevalence is estimated to be 20–25 percent worldwide, as well as 5–18 and 25–31% among populations in Iran and Asian nations, respectively^2–4^. Finding practical methods to prevent and cure NAFLD is essential because it places a heavy financial strain on the healthcare system and lowers quality of life as the illness worsens^5^. It is believed that poor dietary practices, particularly the intake of a high-calorie diet heavy in saturated fatty acids or simple carbohydrates, are mostly to blame for this high prevalence of NAFLD^6^. There is not yet agreement on the pharmacological treatments for NAFLD. The cornerstone of NAFLD therapy is still thought to be lifestyle therapies that emphasize physical exercise and a balanced diet in terms of both quality and quantity^1^.

This treatment's cornerstone is a change in lifestyle that starts with a decrease in the intake of foods that are rich in red meat, trans and saturate fatty acids, processed carbohydrates, and high-fructose corn syrup; low in fiber; and high in energy density^7^. The two well-researched healthy eating patterns (rich in vegetables, fruits, types of antioxidant micronutrients, high fiber, and whole grains) are the Mediterranean and Dietary Approaches to Stop Hypertension (DASH) diets^8^, the effect of which on the reduction in NAFLD odds has been demonstrated^9,10^. A healthy Nordic diet (HND), also known as the Baltic Sea diet, is an additional plant-based dietary pattern that refers to a nutritional profile that is prevalent in the Nordic regions^11^. The HND is rich in high consumption of fruits and vegetables such as cabbage, barriers, legumes and root vegetables, fresh herb, plants and mushrooms, potatoes and nuts, whole grains from oat, repressed oil, and also emphasize the importance on consumption of white and low-fat meat, lower amount of sugar-sweetened products^12^. The composition of food differs between communities, making it difficult to assess connections between specific dietary components such as micronutrients and macronutrients with health outcomes. Dietary scores were developed to include the synergistic effects and combinations of various meals and minerals. They act as a gauge of dietary adherence and indicate a summary value of consumed foods and nutrients^13^. The Baltic Sea Diet Score (BSDS) was developed to quantify a HND based on traditional Nordic foods eaten in Finland. The idea behind the HND prioritize the healthy foods which locally produced, easily accessible, and culturally acceptable. The diet is rich in fruits, barriers, vegetables, low- or non-fat dietary, repressed oil and fish, and low intake of processed meat and alcohol^14^.

The diet is high in fruits consumption, nuts, vegetables, low- or non-fat dietary, repressed oil and fish, and low in processed meats and alcohol^14^.

Previous research has demonstrated that a higher BSDS is connected with a lower risk of abdominal obesity, improved physical ability in old age, and a lower risk of increased C-reactive protein levels which are also known as risk factors of NAFLD disease^15–17^. Other comparable diet scores that have been devised to characterize a HND have also been associated with a reduction in disease risk factors. Despite the fact the relationship with risk of illnesses or death are inconsistent^18^.

Therefore, due to rising frequency of NAFLD in communities and limited data about adherence to HND and the risk of NAFLD, we aimed to examine whether following HND, based on BSDS and Healthy Nordic Diet Index (HNDI) was related with risk of NAFLD among Iranian adults.

## Methods

### Study design and population

This study encompassed a case–control design conducted from 2020 to 2022, involving individuals aged 18 and above who were recently diagnosed with NAFLD. The healthy control group consisted of individuals admitted to Taleghani Hospital in Tehran, Iran, and the academic liver disease clinics of Shahid Sadoughi University of Medical Sciences in Yazd, Iran. The case group included 552 consecutive patients diagnosed with NAFLD by a gastroenterologist. The control group consisted of 340 people without a previous history of NAFLD, who were recruited from the same hospital. The patient sampling technique was validated by two dietitians. The following criteria were used in the diagnosis of NAFLD^19–21^: Chronic elevation of liver enzymes, defined as liver enzymes exceeding 19 U/L for women and 30 U/L for men, along with abstention from alcohol consumption, ultrasonography (US) results indicating NAFLD, liver biopsy findings consistent with NAFLD (Grades II and III), and the exclusion of alternative causes of liver disease. Furthermore, the individuals comprising the case group were directed to our medical facilities for assessment using Fibroscan^19^. The confirmation of non-alcoholic steatohepatitis diagnosis was conducted by a gastroenterologist upon observing Fibroscan results indicating a controlled attenuation parameter score exceeding 237 and a fibrosis score surpassing 7. Additionally, the control group, consisting of individuals without a history of NAFLD, was recruited from several outpatient clinics within the same hospital, including dermatology, ophthalmology, and otorhinolaryngology. The healthy control group consisted of individuals who adhered to a regular dietary regimen for a duration of six months before to the study. Additionally, these individuals had no prior medical records indicating the presence of chronic or inflammatory conditions, including but not limited to diabetes, gastrointestinal disorders, cardiovascular disorders, and cancer. The control group's inclusion criteria were determined by laboratory tests and liver ultrasonography to confirm the absence of hepatic steatosis at any stage. Patients that were excluded from the study met the following criteria: Long-term dietary modifications, weight loss, and specific medical conditions, including hepatic or renal diseases (such as nonalcoholic steatohepatitis (NASH), alcoholic fatty liver disease, Wilson's disease, cirrhosis, autoimmune liver disease, hemochromatosis, and viral infections), diabetes, cancer, thyroid disorder, and autoimmune disease, are factors that may require consideration. Demographic, economic, and social questionnaires were used to gather data pertaining to age, degree of education, work status, medical history, smoking status, usage of particular pharmaceuticals (excluding routine NAFLD medications), and dietary history during the preceding six months. The researchers used General Practice Physical Activity Questionnaires (GPPAQs) to assess the participants' levels of physical activity. GPPAQ is a straightforward assessment tool used to evaluate an individual's present level of physical activity^21^. Nutritionists were utilized as interviewers in this research. As a result, all patients completed the survey questions completely.

The present study received approval from Shahid Beheshti University of Medical Sciences in Tehran, Iran, as well as Shahid Sadoughi University of Medical Sciences in Yazd, Iran. We confirm that all methods were performed in accordance with relevant guidelines and regulations and we also confirm that informed consent was obtained from all individuals and/or their guardians or legal guardians.

The minimum required sample size for this study was determined by considering the hypothesis of a 1.5-fold reduction in the odds of NAFLD associated with the intervention of interest. Hence, taking into account a type I error rate of 5%, a study power of 90%, and an approximate ratio of controls to cases of 1.5, the minimum required sample size was calculated to be 450 people in the case group and 300 people in the control group.

### Dietary assessment

Data on dietary consumption during the preceding year were collected using a semi-quantitative validated food-frequency questionnaire (FFQ) consisting of 168 food items^22^. The FFQ included a comprehensive list of typical Iranian foods and their corresponding serving sizes. Participants provided self-reports on the FFQ, indicating the average portion size and frequency of consumption for each food item. The frequency of consumption options ranged from never to daily, with specific categories such as 2–3 times per month, once per week, 2–4 times per week, 5–6 times per week, and daily. Serving quantities were measured in grams using standard Iranian household measurements^23^. Daily nutrient consumptions for each individual were calculated by utilizing the United States Department of Agriculture's (USDA) national nutritional databank^24^. The nutritional and calorie content of the foods were analyzed using a customized version of Nutritionist 4, specifically designed for Iranian meals, developed by First Databank Inc., Hearst Corp., San Bruno, CA, USA.

### Dietary indices

The BSDS has a total of nine parts, as enumerated below. It was developed using the methodologies described in Kanerva et al.^14^: 1 1) Various categories of fruits and berries, vegetables encompassing roots, pulses, and other varieties, cereals excluding rice and pasta, fish, both processed and unprocessed meat, low-fat or fat-free milk, 7) the ratio of polyunsaturated fatty acids (PUFAs) to saturated fatty acids (SFAs) and trans fatty acids, 8) Alcohol has been excluded from the BSDS due to its prohibition in Iran, 9) Additionally, the BSDS considers total fat and its proportion as a percentage of total energy intake, as indicated in point eight. The intake of participants was used to classify each component of the BSDS into tertiles (Q1-Q3). In the case of healthy items, scores of 1, 2, and 3 were assigned to tertiles Q1 to Q3, respectively. However, for harmful items, namely meat and total fat, the scoring system was reversed. The Baltic Sea Diet adherence is indicated by a higher BSDS score, which spans from 0 to 24 points.

The first estimation of the Healthy Nordic Diet Index (HNDI) was conducted by Olsen et al.^25^ using a set of six items. These items were fish, cabbage, vegetables, whole grains, oats, apples, pears, fruits with high antioxidant activity, and root vegetables. In this index, the six components are categorized into tertiles (Q1-Q3) according to the individuals' intake levels. Subsequently, the tertiles ranging from Q1 to Q3 are assigned scores of 1, 2, and 3, respectively. The level of adherence exhibited a range of values between 0 and 18, with higher values of HNDI indicating more adherence.

### Anthropometric measurement

The researchers conducted an anthropometric study. The weight measurements were obtained by using an SECA 700 Digital Scale (SECA, Hamburg, Germany), which is a standard instrument often used for this purpose. The measurements were rounded to the nearest 100 g. Participants were instructed to wear minimal clothes and remove their shoes before to being weighed. The height of the patient were assessed using a Seca portable height gauge that had an accuracy of 0.1 cm. Furthermore, the researchers used a Seca waist measuring instrument to determine the waist circumference (WC) across the central region spanning from the iliac crest to the last rib. Furthermore, the measurement of hip circumference was obtained in cm by positioning a measuring tape parallel to the floor at the point of maximum fullness of the buttocks. The calculation of body mass index (BMI) included dividing the weight (in kilograms) by the square of the height (in meters), as per the previously described procedure. The researcher performed anthropometric assessments in order to minimize observational variation.

### Biochemical measurement

The laboratory technician collected 10 ml of venous blood from the participants at the commencement and conclusion of the study, after a fasting period of 10–12 h. Following the occurrence of clotting in the surrounding environment, the serum was expeditiously isolated using the process of centrifugation and then preserved at a temperature of − 70 °C till its transportation to the laboratory for the purpose of conducting tests. The concentrations of triglycerides (TG), high-density lipoprotein cholesterol (HDL-C), and fasting blood glucose (FBG) were measured using an enzymatic colorimetric approach using a kit provided by Pars Azmon Company, located in Tehran, Iran. The total cholesterol content was determined by enzyme photometry using the Pars test kit (Parsazmun, Tehran, Iran). The concentration of low-density lipoprotein cholesterol (LDL-C) was measured using the Friedewald formula^26^. LDL-C concentration was also calculated using Friedewald formula: LDL-C (mg/dL) = TC (mg/dL) − HDL-C (mg/dL) − TG (mg/dL)/5. Based on an automated analysis conducted using the BT-3000 system. The measurement of alanine aminotransferase (ALT) and aspartate aminotransferase (AST) enzymes was conducted using enzymatic reagents that were commercially available from Pars Azmoon in Tehran, Iran.

### Statistical analysis

The statistical analysis was performed using the Statistical Package Software for Social Science v.21 (SPSS Inc., Chicago, IL, USA). The normality of the data was assessed by the use of the Kolmogorov–Smirnov's test and the examination of histogram charts. The study collected data on baseline and dietary intakes, representing quantitative variables as mean standard deviation (SD) and qualitative variables as number and percentages. The independent sample t-tests and chi-squared tests were used to compare data between two groups for continuous and categorical variables, respectively. Logistic regression was used to investigate the association between HNDI and BSDS scores and the risk of NAFLD. The analyses were adjusted for possible confounders, including gender, BMI, WC, hip circumference, physical activity, smoking status, education level, drug usage, history of illness, caloric intake, FBG, ALT, AST, lipid profiles, and dietary fiber. The odds ratio (OR) of NAFLD was computed across quartiles of scores, with a 95% confidence interval (CI). We deemed P-values less than 0.05 to be statistically significant.

### Ethics approval and consent to participate

This study was approved by the research council and ethics committee Shahid Beheshti University of Medical Sciences, Tehran, Iran.

## Results

The average age of the study population was 39.53 ± 9.79 years, as shown by the mean (± standard deviation). The average BMI was 27.10 ± 4.45 kg/m2, with the standard deviation (SD) indicating the variability of the data. The average values for BSDS and HNDI were found to be 16.00 ± 2.49 and 11.99 ± 2.61, respectively.

Table 1 presents an overview of the participants' basic characteristics and biochemical data, categorized according to the quartiles of BSDS and HNDI. There was a substantial rise in the age of individuals belonging to the highest quartiles of BSDS and HNDI in comparison to those in the lowest quartiles. Furthermore, a notable disparity was seen in the educational attainment levels across the quartiles of the examined indices. No statistically significant variations were seen between the quartiles of indices and other factors.

**Table 1:** Socio-demographic characteristics, and anthropometric variables across the Quartiles of Healthy Nordic Diet Index (HNDI) and Baltic Sea Dietary Score (BSDS).

		Quartiles of HNDI		P-value	Quartiles of BSDS		P-value
		Q1	Q4		Q1	Q4	
*Demographic variables*							
Age, y		37.65 (9.74)	40.70 (9.37)	**0.002**	38.49 (10.55)	40.79 (9.63)	**0.047**
Female, n (%)		142 (55.0)	126 (47.0)	0.054	122 (49.8)	134 (53.2)	0.283
BMI, kg/m^2^		26.68 (4.24)	26.21 (4.92)	0.156	26.93 (4.37)	27.61 (4.94)	0.136
Weight, kg		71.89 (12.85)	75.22 (15.94)	0.258	71.47 (12.84)	73.87 (14.13)	0.258
Waist- circumference (cm)		91.34 (12.30)	93.47 (11.81)	0.314	91.95 (13.09)	93.75 (11.39)	0.205
Physical Activity (Met.h/wk)		1516.07 (888.61)	1388.22 (902.50)	0.160	1470.23 (896.01)	1334.80 (857.83)	0.378
Smoking (yes), n (%)		9 (4.1)	3 (1.8)	0.165	4 (2.1)	5 (2.9)	0.458
Disease history (yes), n (%)		17 (6.6)	35 (13.1)	0.055	16 (6.5)	3 (12.7)	0.084
Drug use (yes), n (%)		22 (9.5)	27 (11.65)	0.645	19 (9.2)	21 (10.1)	0.709
Education n (%)	Less than a diploma	46 (17.8)	41 (15.3)	**0.014**	50 (20.4)	38 (15.1)	**0.032**
	Diploma	98 (38.0)	106 (39.6)		82 (33.5)	103 (40.9)	
	Bachelor	77 (29.8)	56 (20.9)		82 (33.5)	56 (22.2)	
	Higher than Bachelor	37 (14.3)	65 (24.3)		31 (12.7)	55 (21.8)	
ALT(mg/dl)		31.43 (40.66)	34.02 (28.63)	0.716	30.63 (37.1)	35.11 (30.31)	0.379
AST(mg/dl)		19.94 (9.06)	25.30 (14.25)	0.125	20.72 (9.42)	25.73 (14.65)	0.051
FBS (mg/dl)		111.69 (30.43)	115.26 (41.72)	0.942	111.15 (28.40)	116.54 (41.22)	0.708
TC (mg/dl)		159.94 (39.92)	179.27 (48.27)	0.415	166.95 (47.30)	182.45 (45.87)	0.485
TG (mg/dl)		148.97 (72.13)	173.71 (81.24)	0.214	166.55 (83.27)	178.65 (84.29)	0.430
LDL-C (mg/dl)		103.25 (22.28)	109.75 (38.47)	0.652	108.40 (31.45)	110.74 (38.76)	0.673
HDL-C (mg/dl)		43.28 (6.15)	45.13 (18.81)	0.546	42.02 (8.40)	43.83 (15.83)	0.573

Values are expressed as means (standard deviation (SD)) of 891 subjects.

P-values are resulted from the student t-test.

Significant values are in bold.

BMI, Body mass index.

Dietary intake of subjects across the quartiles of BSDS and HNDI are presented in Table 2. Compared with those in the lowest quartile of HNDI, subjects in the highest quartile had higher energy, carbohydrate, protein, fat, SFA, MUFA, PUFA, cholesterol, fiber, potassium, iron, calcium, magnesium, zinc, vitamin C, E, D, B9, caffeine and all of food groups. No significant difference was found for sodium across quartiles of HNDI. Also, individuals in the highest quartiles of BSDS had higher intake of energy, carbohydrate, protein, PUFA, fiber, potassium, iron, calcium, magnesium, zinc, vitamin C, E, D, B9, caffeine, total dairy, legume, nut, fish, whole grains, fruits, and vegetables as well as a lower intake of red and processed meat.

**Table 2:** Dietary intake across the Quartiles of Healthy Nordic Diet Index (HNDI) and Baltic Sea Dietary Score (BSDS).

	Quartiles of HNDI		*P *value	Quartiles of BSDS		*P *value
	Q1	Q4		Q1	Q4	
*Dietary intake*						
Energy (Kcal/d)	1837.07 (540.45)	2580.77 (596.95)	** < 0.001**	1960.12 (587.08)	2416.00 (636.38)	** < 0.001**
Carbohydrate (g/d)	260.21 (85.78)	377.31 (97.54)	** < 0.001**	259.64 (84.90)	367.16 (104.94)	** < 0.001**
Protein (g/d)	62.10 (21.51)	97.94 (30.46)	** < 0.001**	68.31 (25.02)	90.17 (30.96)	** < 0.001**
Fat (g/d)	64.89 (25.08)	87.19 (28.71)	** < 0.001**	77.05 (28.24)	26.18 (26.27)	0.619
SFA (g/d)	21.59 (9.75)	27.93 (10.40)	** < 0.001**	25.96 (10.41)	23.96 (9.00)	0.129
MUFA	22.39 (9.01)	28.64 (10.66)	** < 0.001**	26.54 (10.13)	25.21 (9.78)	0.292
PUFA	14.15 (6.77)	19.54 (10.00)	** < 0.001**	16.46 (8.03)	17.35 (8.15)	**0.035**
Cholesterol (mg/d)	196.48 (154.85)	301.30 (150.83)	** < 0.001**	230.75 (119.11)	258.98 (143.19)	0.084
Fiber(g/d)	26.70 (19.07)	38.27 (16.51)	** < 0.001**	26.58 (17.75)	38.70 (19.99)	** < 0.001**
Sodium (mg/d)	3964.78 (4082.73)	4296.38 (2892.50)	0.456	4172.10 (4382.61)	4160.41 (2944.57)	0.536
Potassium (mg/d)	2547.18 (888.96)	4670.14 (1235.89)	** < 0.001**	2768.81 (967.40)	4354.29 (1313.20)	** < 0.001**
Iron (mg/d)	25.23 (52.61)	40.37 (41.61)	** < 0.001**	23.25 (26.60)	37.84 (36.72)	**0.001**
Calcium (mg/d)	910.99 (435.00)	1239.06 (469.08)	** < 0.001**	954.37 (446.00)	1185.98 (450.24)	** < 0.001**
Magnesium (mg/d)	261.65 (94.71)	413.46 (118.03)	** < 0.001**	269.28 (92.07)	401.18 (132.78)	** < 0.001**
Zinc(mg/d)	8.69 (2.97)	12.79 (3.45)	** < 0.001**	9.42 (3.15)	11.84 (3.78)	** < 0.001**
Vitamin C(mg/d*)*	87.36 (53.80)	220.44 (97.710	** < 0.001**	98.86 (63.08)	205.00 (97.76)	** < 0.001**
Folate (mcg/d)	403.28 (171.88)	517.76 (180.57)	** < 0.001**	397.20 (170.09)	507.02 (183.67)	** < 0.001**
Vitamin E (mg/d)	9.06 (4.30)	11.92 (4.94)	** < 0.001**	9.75 (4.79)	11.17 (4.79)	**0.002**
Vitamin D (mcg/d)	1.31 (1.30)	2.01 (1.91)	** < 0.001**	1.44 (1.36)	1.84 (1.48)	**0.001**
Caffeine (mg/d*)*	104.78 (69.08)	131.29 (112.83)	**0.001**	108.19 (74.43)	129.41 (100.65)	**0.013**
*Food groups*						
Total dairy (g/d)	328.85 (236.67)	412.18 (255.58)	** < 0.001**	349.71 (259.32)	402.20 (247.63)	0.075
Legume (g/d)	9.04 (8.27)	32.97 (28.14)	** < 0.001**	16.22 (19.51)	25.32 (25.89)	** < 0.001**
Nut (g/d)	4.78 (6.04)	12.16 (14.94)	** < 0.001**	5.90 (8.880	10.90 (12.62)	** < 0.001**
Fish (g/d)	5.50 (5.44)	16.44 (20.20)	** < 0.001**	6.36 (6.21)	14.77 (12.02)	** < 0.001**
Whole grains (g/d)	55.46 (69.74)	108.87 (83.40)	** < 0.001**	46.53 (53.97)	120.67 (109.11)	** < 0.001**
Refined grains (g/d)	328.97 (205.26)	277.64 (170.29)	**0.008**	299.06 (191.52)	278.94 (175.43)	0.250
Red and Processed meat (g/d)	26.58 (27.69)	43.78 (36.00)	** < 0.001**	39.38 (32.84)	30.60 (31.24)	**0.020**
Fruits (g/d)	240.42 (209.11)	675.02 (403.74)	** < 0.001**	262.67 (221.52)	630.69 (374.31)	** < 0.001**
Vegetables (g/d)	189.62 (111.60)	399.78 (165.94)	** < 0.001**	211.35 (127.93)	381.68 (172.89)	** < 0.001**

Values are expressed as means (standard deviation (SD)) of 891 subjects.

P-values are resulted from the student t-test.

Significant values are in bold.

The odds ratios (ORs) and 95% confidence intervals (CIs) for individuals with NAFLD are shown in Table 3, categorized according to quartiles of BSDS and HNDI.

**Table 3:** Odds ratio (OR) and 95% confidence interval (CI) for NAFLD based on Healthy Nordic Diet Index (HNDI) and Baltic Sea Dietary Score (BSDS).

	Quartiles of scores				*P* for trend
	Q1	Q2	Q3	Q4	
*HNDI*					
Crude model	1.00 (Ref)	0.69 (0.42–1.13)	0.93 (0.63–1.38)	0.99 (0.67–1.47)	0.870
Model 1*	1.00 (Ref)	0.64 (0.39–1.05)	0.88 (0.59–1.31)	0.95 (0.64–1.41)	0.957
Model 2^†^	1.00 (Ref)	0.65 (0.27–1.59)	0.56 (0.26–1.19)	0.42 (0.18–0.98)	**0.043**
*BSDS*					
Crude model	1.00 (Ref)	0.92 (0.61–1.38)	0.70 (0.42–1.16)	0.49 (0.31–0.77)	**0.001**
Model 1*	1.00 (Ref)	0.89 (0.59–1.34)	0.67 (0.40–1.12)	0.46 (0.29–0.73)	**0.001**
Model 2^†^	1.00 (Ref)	0.88 (0.52–1.36)	0.63 (0.39–1.16)	0.48 (0.32–0.89)	**0.003**

**Binary logistic regression was used to obtain OR and 95% CI.

*Model 1: adjusted for age and sex.

^†^Model 2: Model 1 + BMI, Waist circumference, hip circumference, physical activity, smoking, education, drug use, disease history, FBS, ALT, AST, Lipid profiles, fiber, and energy intake.

Significant values are in bold.

In the crude and initial adjusted model, which accounted for age and sex, no statistically significant association was found for HNDI in the highest quartile compared to the lowest quartile (odds ratio [OR] = 0.99, 95% confidence interval [CI] 0.67–1.47; p for trend = 0.870; OR = 0.95, 95% CI 0.64–1.41; p for trend = 0.957, respectively). Nevertheless, when controlling for confounding variables using the final model, it was seen that increased adherence to the HNDI was associated with a reduced likelihood of NAFLD (odds ratio [OR]: 0.42; 95% confidence interval [CI]: 0.18–0.98; p for trend = 0.043). A notable association was observed between a decrease in the likelihood of NAFLD among individuals with the highest score of BSDS, in comparison to those with the lowest score. This association was evident in both the unadjusted model (odds ratio [OR] = 0.49, 95% confidence interval [CI] 0.31–0.77; p-value for trend = 0.001) and the model adjusted for confounding factors (OR = 0.48, 95% CI 0.32–0.89; p-value for trend = 0.003).

## Discussion

The association between HND and the risk of NAFLD was investigated in this research. After adjusting for age, sex, BMI, WC, hip circumference, physical activity, smoking, education, drug use, illness history, FBS, ALT, AST, Lipid profiles, fiber, and calorie consumption, it is noteworthy that individuals with higher BSDS and HNDI scores had decreased probabilities of developing NAFLD. To the best of our knowledge, no prior research have indicated a relationship between HND and NAFLD chances.

Indeed, dietary patterns such as excessive calorie intake, high fructose consumption, and insufficient physical exercise are the most important risk factors for NAFLD. Previous studies demonstrated that the Mediterranean diet have positive benefits on reducing the NAFLD odds^9^. Despite the fact that the items in Mediterranean diet and HND belong to distinct varieties, there are commonalities between them, such as the fact that all of them are rich in fruits, vegetables, whole grains, fish, and low-fat dairy products. Furthermore, both of these diets have been linked to a decreased risk of various disorders, including diabetes and cardiovascular disease, as well as alterations in body homeostasis, such as insulin resistance and inflammation^27^. Because of this, we consider that feasible explanation for the observed relationships between the healthy diet and NAFLD are multifactorial, such as the favorable effects of diet on risk factors for chronic illnesses.

Inflammation and oxidative stress are the primary contributors to the pathophysiology of NAFLD^1^. Hence, the other important risk factors are being overweight or obese, having diabetes, having hyperlipidemia, not getting enough exercise, and eating an unhealthy diet^28–30^. Multiple pathways may have mediated the effective benefits of a HND with NAFLD risk factors.

Several processes, including alterations in cytokines, inflammatory factors, insulin resistance, and dyslipidemia, have been reported to explain the link between obesity and fatty liver^31^, and also up-regulating the expression of some of the genes in the liver of obsess patients diagnosed with NAFLD were founded^32^. For instance, the HND has been demonstrated to reduce the probability of obesity^15^. Besides, Kolehmainen et al. declared that a HND decreases inflammatory gene expression in SAT when compared to a control diet, regardless of changes in body weight in the patients with a metabolic syndrome^33^. A recent meta-analysis also illustrated that adherence to HND meaningfully reduce body weight^34^.

Moreover, a large Danish cohort research revealed an adverse relationship between adherence to HND and low risk of T2D^35^. Interestingly, in patients with hypercholesterolemia, a HND improves blood lipid profile and insulin sensitivity while also lowering blood pressure to clinically meaningful levels^36^ In the randomized dietary study, participations with metabolic syndrome had significant changes in non-HDL-C , LDL-C to HDL-C ratio, and Apo B to Apo A1 ratio, which has been reported adherence to HND improved lipid profile^37^. By contrast, we demonstrated that changing in lipid profile were not significantly different between the groups.

The favorable benefits of the HND on NAFLD may be attributed to several reasons. The Nordic dietary pattern emphasizes eating foods with high content of fiber and are linked to a greater sense of fullness^38^. A high concentration of soluble and insoluble fibers is related to a reduction in serum TAG and blood glucose^39^.Indeed, this diet may be beneficial for NAFLD patients.

It is noteworthy to notice that the two scores to represent a HND, HNDI and BSDS, which were formed fairly differently from one another, provided consistent relationships with the risk of developing NAFLD. The HNDI was based on intakes of fish, apples and pears, and root vegetables, cabbage, oatmeal, rye bread, however, it did not include some of the factors that were used in the BSDS. These factors included dietary fat quality as well as dairy and meat intakes^40^.

The strengths of the present study include a large study population consisting of both men and women, comprehensive data about potential cofounding factors and also no loss in follow-up. A trained interviewer was filled out the questionnaires in order to minimizing the random errors in recording. Our study had some potential limitations, there is a risk of recall bias in the present study due to the retrospective way of the data collection. Hence, to reduce the bias, a valid and trustworthy FFQ was utilized. It also seems that the level of adherence to this type of dietary index in countries and regions (Baltic countries) is different compared to the region where the study was conducted (Iran) and this itself can be a limitation.

In conclusion, our findings revealed that adherence to a healthy Nordic diet remarkably reduces the risk of developing NAFLD, demonstrating that the highly nutritious components of the Nordic diet are beneficial for reducing the risk of NAFLD. Therefore, a high adherence to healthy Nordic dietary pattern may be effective also in reducing the possibility of developing the NAFLD risk factors. Further longitudinal studies in diverse population are warranted to confirm our results.

## Data Availability

Data are available upon request from the corresponding author (Mohammad Hassan Sohouli) due to privacy/ethical restrictions.
